# Investigator initiated clinical trial to validate usefulness of specific system for endoscopic ultrasound guided hepaticogastrostomy (HG01) in malignant biliary obstruction (HG01)

**DOI:** 10.1097/MD.0000000000029408

**Published:** 2022-06-03

**Authors:** Masahiro Itonaga, Masayuki Kitano, Hiroyuki Isayama, Mamoru Takenaka, Takeshi Ogura, Yasunobu Yamashita, Toshio Fujisawa, Kosuke Minaga, Atsushi Okuda, Toshio Shimokawa

**Affiliations:** a Second Department of Internal Medicine, Wakayama Medical University, Wakayama, Japan; b Department of Gastroenterology, Graduate School of Medicine, Juntendo University, Tokyo, Japan; c Department of Gastroenterology and Hepatology, Kindai University Faculty of Medicine, Osaka, Japan; d The Second Department of Internal Medicine, Osaka Medical and Pharmaceutical College, Takatsuki, Japan; e Clinical Study Support Center, Wakayama Medical University, Wakayama, Japan.

**Keywords:** endoscopic ultrasound-guided biliary drainage (EUS-BD), endoscopic ultrasound-guided hepaticogastrostomy (EUS-HGS), malignant biliary obstruction

## Abstract

**Introduction:**

Endoscopic ultrasound-guided hepaticogastrostomy (EUS-HGS) is a novel drainage option for patients with an inaccessible papilla. Although EUS-HGS has clinical benefits in patients for whom endoscopic retrograde cholangiopancreatography (ERCP) has failed, the rates of adverse events (AEs) associated with EUS-HGS, such as bile peritonitis and stent migration, are higher than for other procedures. The development of a dedicated system for EUS-HGS is therefore desirable to reduce the rate of AEs. We developed a dedicated system for EUS-HGS (HG01 system) which is composed of a 19-gauge needle, 0.025-inch guidewire, a thin delivery system for tract dilation, and an antimigration metal stent. This study is designed to evaluate the efficacy and safety of EUS-HGS using the HG01 system in malignant biliary obstruction.

**Methods/design:**

This is a single-arm multicenter prospective study involving 40 patients across six tertiary centers in Japan. Patients with an unresectable malignant biliary obstruction in whom biliary drainage with ERCP failed, is not possible, or is very difficult will be registered in the study. The primary endpoint is the clinical success rate. The secondary endpoints are the technical success rate, procedure-related AE rate, procedure time, procedure success rate using only the HG01 system, stent patency rate, re-intervention success rate, re-intervention method, survival rate, and distance of movement of the stent position.

**Discussion:**

We expect use of the HG01 system to reduce the rate of AEs during EUS-HGS, especially bile leakage and stent migration. If the efficacy and safety of EUS-HGS using the HG01 system is confirmed in the present study, it is likely to be considered the first-choice device for use during EUS-HGS.

## 1 Introduction

Endoscopic ultrasound-guided biliary drainage (EUS-BD) was first reported in 2001^[1]^ and is considered to be an effective drainage method for patients with bile duct obstruction for whom biliary drainage by endoscopic retrograde cholangiopancreatography (ERCP) is unsuccessful or difficult.^[2–4]^ Compared with percutaneous transhepatic biliary drainage, which has been used when ERCP is unsuccessful, EUS-BD can be performed as an internal drainage method and has a higher technical success rate (100% vs 86.4%, *P* < .007) and lower adverse event (AE) rate (18.2% vs 70.6%, *P* < .001).^[5]^ We previously conducted a multicenter prospective clinical study to evaluate the efficacy and safety of EUS-BD using a metal stent for ERCP and reported on its efficacy and safety.^[6,7]^ Moreover, compared with conventional drainage under ERCP, EUS-BD is expected to have a longer patency period because the stent is not located within the tumor.^[8]^

EUS-BD can be categorized into two main types: EUS-guided hepaticogastrostomy (EUS-HGS), which creates an anastomosis between the stomach and the intrahepatic bile duct, and EUS-guided choledochoduodenostomy (EUS-CDS), which creates an anastomosis between the duodenal bulb and the extrahepatic bile duct. EUS-CDS can be indicated in patients with distal biliary obstruction and normal gastrointestinal structures, whereas EUS-HGS is more widely indicated in patients who have hilar biliary obstruction and/or surgical reconstruction. However, the rates of AEs associated with EUS-HGS, such as bile peritonitis and stent migration, are higher than for EUS-CDS.^[9]^ One of the reasons for the high rate of AEs associated with EUS-HGS is that no dedicated devices are available for EUS-HGS, with devices and metal stents designed for ERCP being used instead. EUS-HGS using these devices and metal stent has been reported to be associated with bile peritonitis due to stent migration.^[10]^ In addition, the leakage of bile during fistula dilation step also causes postprocedural bile peritonitis.^[11,12]^ The development of a dedicated antimigration metal stent with a thin delivery system for tract dilatation for EUS-HGS is therefore desirable to reduce the rate of AEs.

We thus developed a partially covered self-expandable metal stent with a thin delivery system (7.2F) dedicated to EUS-HGS and reported on the efficacy and safety of EUS-HGS in experimental settings.^[13]^ It is expected that EUS-HGS with the dedicated delivery system will reduce bile leakage by eliminating the dilation step and the dedicated stent with laser-cut wire and anchoring hooks may help to prevent stent migration. Moreover, we also developed other dedicated devices specific to EUS-HGS such as a 19-gauge needle and 0.025-inch guidewire. This single-arm multicenter study aims to evaluate the efficacy and safety of EUS-HGS using the dedicated system (HG01 system) in patients with malignant biliary obstruction after failed ERCP.

## 2 Methods/design

### 2.1 Ethical approval and patient consent

This study was approved by the Wakayama Medical University Hospital Institutional Review Board (number: 1-02021A) and, subsequently, by the institutional review boards of all other participating centers. All patients will provide written informed consent. The trial is registered with the Japan Registry of Clinical Trials (JRCT; trial registration: jRCT2052210020).

### 2.2 Study aims and design

This is a single-arm multicenter study across six tertiary centers in Japan that aims to evaluate the efficacy and safety of a system for endoscopic ultrasound-guided hepaticogastrostomy (HG01) in 40 patients with malignant biliary obstruction. The first three patients will be performed at the main institution, Wakayama Medical University. An interim analysis of safety will be performed after the procedure is completed on the first three patients, and the Data and Safety Monitoring Committee (DSMC) will evaluate the safety of the system and decide whether the trial can be continued afterwards. If the DSMC decides that the system is safe enough to continue the trial, the trial will be conducted in the remaining 37 patients at all participating institutions.

### 2.3 Patients

At each center, the on-site study investigators will obtain informed consent from the candidates, input necessary information into an electronic data capture system, confirm that the candidates meet the eligibility criteria (i.e., the candidates meet all the inclusion criteria and none of the exclusion criteria), and register the candidates with the registration secretariat. After confirming that a candidate meets the criteria, a registration number will be issued, and the registration will be considered complete.

### 2.4 Inclusion criteria

The following inclusion criteria will be applied.

(1) Age of more than 20 years when providing informed consent.
(2) Pathological diagnosis of malignancy.
(3) Biliary obstruction due to unresectable malignant tumor.
(4) Biliary drainage with ERCP failed, was not possible, or was considered difficult.
(5) Serum bilirubin level of higher than 3.0 mg/dL before enrollment.
(6) Informed consent document obtained from the patient.

### 2.5 Exclusion criteria

The following exclusion criteria will be applied.

(1) Impossible to perform endoscopy.
(2) Total gastrectomy has already been performed.
(3) Left hepatectomy has already been performed.
(4) Bismuth classification for hilar biliary carcinoma: Type IIIb or Type IV.
(5) Liver cirrhosis.
(6) Ascites around the liver on CT.
(7) With percutaneous trans-hepatic biliary drainage (PTBD) tube at time of obtaining informed consent.
(8) Planned insertion of a metal stent in the upper gastrointestinal tract within 7 days before treatment.
(9) Performance status 4.
(10) Other severe diseases of the heart, lung, kidney, and/or liver of ASA 4 or higher.
(11) Poor general condition and estimated prognosis of less than 2 months.
(12) Hemorrhagic diathesis including fatal aneurisms or severe coagulation disorder.
(13) Participation in other clinical trials (except in a non-intervention arm).
(14) Women who may possibly be pregnant or who want to be pregnant.
(15) Metal allergy to nickel-titanium.
(16) Known allergy to iodine contrast agents.
(17) Patients whom the investigators otherwise decide are inappropriate to participate in the trial.

### 2.6 HG01 system

**Figure 1:**
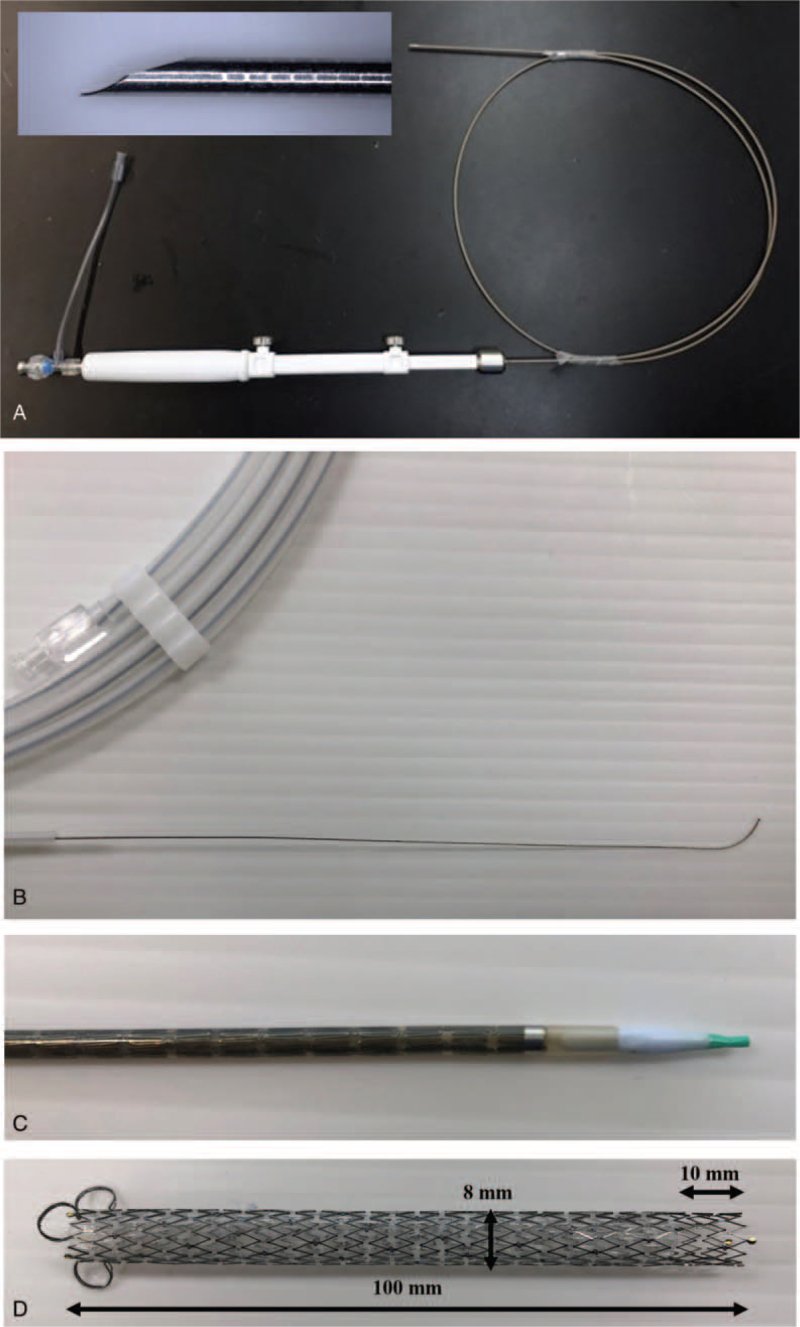
The dedicated system for EUS-HGS (HG01 system). (A) The 19-gauge needle is used to puncture and access the intrahepatic bile duct from the stomach under echoendoscopy and allows insertion of the guidewire with simultaneous injection of the contrast agent. (B) The tip of the guidewire is made of a metal coil, which is less likely to shear because of contact with the needle tip. (C) The diameter of the delivery system is 7.2F, and its tip is soft and tapered. (D) The stent is 8 mm in diameter and 100 mm long. It is made from laser-cut nitinol wire and is partially covered by a polyurethane membrane. The distal 10 mm of the stent is uncovered, and hooks are attached to the proximal end to prevent distal stent migration.

The HG01 system is composed of a 19-gauge needle, 0.025-inch guidewire, delivery system, and metal stent. The needle is used to puncture and access the intrahepatic bile duct from the stomach under echoendoscopy and allows simultaneous insertion of the guidewire with injection of the contrast agent (Fig. 1A). The tip of the guidewire is made of a platinum-iridium metal coil without resin coating, which is less likely to shear because of contact with the needle tip (Fig. 1B). However, this guidewire cannot be used with electrocautery catheter due to its high electric conduction. The delivery system has a diameter of 7.2F and a soft tip that is tapered, which facilitates its insertion into the intrahepatic bile duct without fistula dilation. In experimental settings, the delivery system can be inserted into the bile duct without fistulous tract dilation^[13]^ (Fig. 1C). The design of the stent may prevent stent migration because it is equipped with the partially covered laser-cut structure and antimigration anchoring hooks^[13]^ (Fig. 1D).

### 2.7 Procedure

An echoendoscope is inserted orally and advanced to the stomach. An appropriate target intrahepatic bile duct is then confirmed with EUS from the stomach, and Doppler imaging is used to rule out any intervening vessels. The 19-gauge dedicated needle is then used to puncture the intrahepatic bile duct under endosonographic guidance. After the bile juice is aspirated, contrast medium is injected, and the 0.025-inch dedicated guidewire is advanced into the intrahepatic bile duct. If the guidewire does not reach the downstream part of the bile duct with a long distance sufficient for metallic stent placement, a catheter or another guidewire will be used to reach the bile duct site. Thereafter, an attempt is made to insert the dedicated delivery system via the fistula without using any dilation devices. If this is successful, the dedicated metal stent is expanded between the intrahepatic bile duct and the stomach under EUS and fluoroscopic guidance. If it is difficult to advance the delivery system via the fistula, the fistulous tract is dilated using dilation devices.

### 2.8 Primary endpoint

The primary endpoint is the clinical success rate, which is defined as follows:

(1) Improvement in the serum total bilirubin (T-Bil) level to below 50% of the highest pretreatment level on at least two of four measuring points including days 3, 7, 14, and 21.
(2) A decrease in the T-Bil level after treatment compared with the highest pretreatment T-Bil level, and a decrease in the diameter of the bile duct by 50% or more on day 14 on computed tomography (CT) imaging compared with the largest pretreatment diameter of the bile duct.

### 2.9 Secondary endpoints

The secondary endpoints are as follows:

(1) Technical success rate
  Technical success is defined as the stent being positioned between the stomach and intrahepatic bile duct, as seen on CT performed 1 day after treatment.
(2) Procedure-related adverse event (AE) rate
  Procedure-related AEs are defined as AEs judged to be “related,” “possibly related,” or “might be related” to the procedure. Procedure-related AEs will be divided into early AEs occurring within 14 days after treatment and late AEs occurring from 15 to 60 days after treatment. Early AEs may include postprocedural pancreatitis, cholangitis, stent migration, stent deviation, gastric ulcer associated with mechanical irritation of the stent, bleeding, gastrointestinal injury, and procedure-related deaths. Late AEs may include stent migration, stent deviation, stent occlusion, and gastric ulcer associated with mechanical irritation from the stent.
(3) Procedure time
  Procedure time is defined as the time from the puncture of the bile duct to stent placement.
(4) Procedure success rate using only the HG01 system
  Procedure success using only the HG01 system is defined as successful insertion of the stent into the left intrahepatic bile duct using only the HG01 system.
(5) Stent patency rate at 30 and 60 days after treatment
  The stent patency rates are defined as the percentage of patients who do not experience stent dysfunction from the date of stent insertion to 30 and 60 days after treatment.
  Stent dysfunction is defined as follows.
  (1) A T-Bil level of ≥3.0 mg/dL when the lowest T-Bil level measured after treatment is <1.5 mg/dL.
  (2) A T-Bil level more than 2.0 times the lowest level when the lowest T-Bil level measured after treatment is more than 1.5 mg/dL.
(6) Re-intervention success rate and re-intervention method
  Re-intervention success is defined as the placement of a new drainage tube and improvement in the T-Bil level to below 50% of the highest pretreatment level within 21 days after re-intervention.
  The following re-intervention methods can be selected.
  (1) Treatment from the stent
  (2) Other EUS-guided biliary drainage
  (3) Trans-papillary biliary drainage
  (4) Percutaneous transhepatic biliary drainage
  (5) Surgical cholangiojejunostomy
(7) Survival rate at 60 days after treatment
(8) Distance of movement of the stent position at 1, 14, 30, and 60 days after treatment
  The length of the stent inside the stomach on the abdominal CT at day 0 after treatment will be compared with the length of the stent inside the stomach on days 1, 14, 30, and 60 after treatment, to evaluate the distance of movement of the stent from its placement position.

### 2.10 Endpoints for the interim analysis

An interim safety analysis will be performed at 30 days after study treatment of the third included patient, and the safety of continuing the trial will be evaluated by the Data and Safety Monitoring Committee. If two of the three patients develop a serious procedure-related AE (severe or fatal according to ASGE [American Society for Gastrointestinal Endoscopy] guidelines,^[14]^ or grade 4 or 5 according to CTCAE [Common Terminology Criteria for Adverse Events]), the study will be discontinued.

### 2.11 Data collection

Baseline assessment will be performed during the screening period. Basic information including sex, age, date of birth, primary disease and date of first diagnosis, history, concomitant disease, and metal allergy to nickel-titanium will be recorded. To ensure feasibility and safety, complete blood counts, hepatic and renal function tests, and biochemical tests will be performed during the screening period.

**Figure 2:**
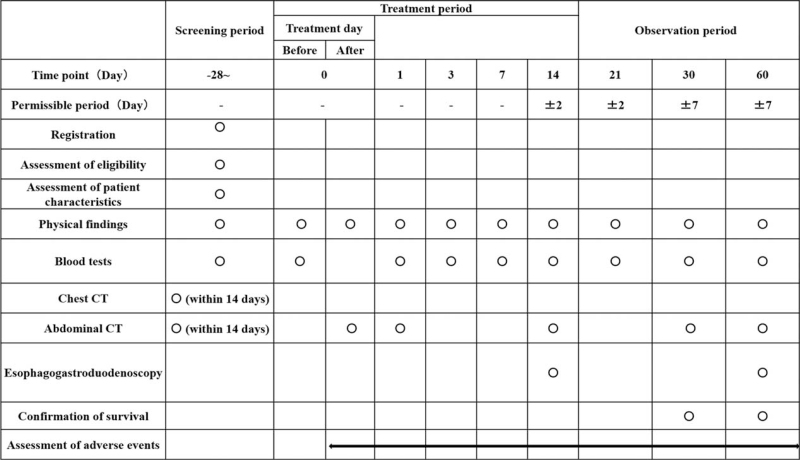
Standard protocol items (SPIRIT): schedule for data collection.

CT will be performed during the screening period to determine the site of biliary obstruction and the tumor stage according to the TNM classification.^[15]^ Patients will undergo the above-mentioned blood tests on postprocedural days 1, 3, 7, 14, 21, 30, and 60 to evaluate the efficacy of the treatment, AEs, and stent dysfunction. CT will be performed postoperatively on days 0, 1, 14, 30, and 60 to evaluate the position of the stent, AEs, and stent dysfunction. Esophagogastroduodenoscopy will be performed postoperatively on days 14 and 60 to evaluate gastric mucosal damage by the stent. All patients will be followed until either death or 60 days after the treatment to record survival, concomitant medications, and concomitant therapy. The schedule for enrollment, interventions, and assessments is shown in Figure 2.

### 2.12 Statistical analysis

#### 2.12.1 Sample size calculation

In a meta-analysis on EUS-BD, the median rate of clinically effective improvement in 26 articles enrolling more than ten patients was 88.0%, and the median of 3 articles enrolling more than 100 patients was 89.5%.^[3]^ In addition, the median rate of clinically effective improvement in a randomized controlled trial was 87.5%.^[16]^ On the basis of these reports, we set the expected clinical response rate for this study to 88%. In the meta-analysis,^[3]^ the 5% point improvement in clinical efficacy of the 26 articles with more than ten cases was 72.5%. Therefore, we set the threshold for the clinically effective improvement rate to 73%. In this case, the minimum number of patients required to achieve a power of 80% or higher at the significance level of α-0.05 is 37 patients. We set the target number of patients to 40, assuming that a small number (about 10%) of patients will drop out of the study.

#### 2.12.2 Statistical analysis

In the effectiveness analysis for the population, the full analysis set (FAS) is the main analysis, and the per protocol analysis set (PPS) acts as a reference. In the safety analysis for the target population, all test examples will be analyzed. For the clinical success rate, a test of the mother ratio against the threshold clinical success rate of 0.73 will be performed for the FAS. The exact method will be used to calculate the *P*-value, and a one-sided test will be performed. If the *P*-value is below the significance level of α = 0.05, EUS-HGS using the HG01 system will be considered effective. For the rates of technical success, procedure-related AE, successful procedures using only the HG01 system, and stent patency at 30 days, the percentages and 95% Clopper–Pearson confidence intervals will be calculated. For the procedure time, the mean and 95% confidence interval will be calculated. For the overall survival time, the survival rate will be estimated using the Kaplan–Meier method, and the 95% confidence interval at each time point will be calculated using Greenwood's formula. In addition, a Kaplan–Meier plot will be used to draw the survival curve. For the distance of movement of the stent position at 1, 14, 30, and 60 days after treatment, the mean and 95% confidence interval will be calculated. We will also create transition charts for each case and for the mean values. Error bars representing standard error will be added to the mean trend chart. The number and percentage of AEs will be calculated for each grade of AEs. All analyses will be performed using R version 4.1.2 (The R Foundation for Statistical Computing, Vienna, Austria).

#### 2.12.3 Monitoring

Visit monitoring will be performed periodically by an independent data monitoring committee. The monitoring committee will collect information on the status of patient accumulation, inclusion/exclusion criteria, serious AEs, and any other relevant information and strive to provide feedback to participating institutions for early resolution if there are any problems. The monitoring committee will also report any serious AEs to the committee for efficacy and safety assessment.

#### 2.12.4 Audit

The audit staff will evaluate whether the clinical trial is conducted in compliance with the Act on Securing Quality, Efficacy and Safety of Products including Pharmaceuticals and Medical Devices; good clinical practice; and all applicable regulatory requirements. They will assure the quality of the clinical trial independently and separately from the normal monitoring and quality control operations of the clinical trial.

## 3 Discussion

This single-arm multicenter study is designed to evaluate the efficacy and safety of EUS-HGS using the HG01 system in malignant biliary obstruction. Because of the use of the new device, an interim evaluation will confirm safety and determine whether the study can be continued afterwards. The primary endpoint of this study is the clinical success rate, while the secondary endpoint is to evaluate the feasibility and safety of EUS-HGS using this device, including the procedure time, procedure-related AE rate, and distance of movement of the stent position after treatment. In addition, since the four devices included in the HG01 system are specific to EUS-HGS, we will also examine whether EUS-HGS can be completed with only these devices.

A meta-analysis demonstrated technical and clinical success rates of 95% and 92%, respectively, for EUS-HGS, while the procedure-related AE rate was 23%. The procedure-related AEs included stent migration (13%), bleeding (17%), pneumoperitoneum (13%), bile leakage (17%), cholangitis (10%), perforation (2%), abdominal pain (7%), and others (21%).^[3]^ Among the various procedure-related AEs, bile leakage and stent migration can develop as serious AEs. The dilation step using a mechanical dilator or cautery device can cause bile leakage leading to the development of bile peritonitis.^[11]^ Also, if the luminal side of the stent moves into the abdominal cavity it can cause fatal bile peritonitis.^[10]^ The development of a dedicated system for EUS-HGS is desirable to reduce the rate of AEs, especially bile leakage and stent migration.

The newly developed dedicated system for EUS-HGS (HG01 system) is composed of a 19-gauge needle, 0.025-inch guidewire, delivery system, and metal stent. EUS-HGS using the HG01 system has the following advantages compared with EUS-HGS using conventional devices. First, it has been reported that when the guidewire is advanced into the intrahepatic bile duct, the tip of the guidewire is sometimes sheared because of contact with the needle tip.^[5,17]^ The tip of the dedicated guidewire is made of a metal coil to avoid the wire being sheared. Second, the diameter of the dedicated delivery system (7.2F) is low to successfully insert it without using other fistulous tract dilation devices, with its tip being soft and tapered. In experimental settings, the dedicated delivery system can be inserted into the bile duct without fistulous tract dilation.^[13]^ It is expected that EUS-HGS with the dedicated delivery system will reduce bile leakage by eliminating the dilation step. Third, the dedicated stent is made from laser-cut wire and has anchoring hooks, features that may help to prevent stent migration.

## 4 Conclusion

This single-arm multicenter study is designed to evaluate the efficacy and safety of EUS-HGS using a dedicated system (HG01 system) for the treatment of malignant biliary obstruction. EUS-HGS using the HG01 system is expected to show a reduced rate of AEs, especially bile leakage and stent migration. If the efficacy and safety of EUS-HGS using the HG01 system is confirmed, it is likely to be considered the first-choice device for use during EUS-HGS.

## Author contributions

MI, MK, HI, MT, TO and TS conceived the study, designed the study protocol, and drafted the manuscript. MI and MK wrote the manuscript. YY, TF, KM and AO are in charge of coordination and direct implementation. TS helped to develop the study measures and analyses. All authors contributed to drafting this study protocol manuscript and have read and approved the final version.

**Conceptualization:** Mamoru Takenaka, Masahiro Itonaga, Masayuki Kitano, Takeshi Ogura, Hiroyuki Isayama.

**Formal analysis:** Toshio Shimokawa.

**Funding acquisition:** Masayuki Kitano.

**Investigation:** Atsushi Okuda, Toshio Fujisawa, Yasunobu Yamashita, Kosuke Minaga.

**Writing – original draft:** Masahiro Itonaga.

**Writing – review & editing:** Masayuki Kitano.
